# Catheter Ablation of Parahisian Premature Ventricular Complexes From the Right Sinus of Valsalva

**DOI:** 10.1111/jce.16513

**Published:** 2024-12-08

**Authors:** Michael Ghannam, Jamie Simpson, Mohamed Al‐Sadawi, Amrish Deshmukh, Jackson J. Liang, Rakesh Latchamsetty, Thomas Crawford, Krit Jongnarangsin, Hakan Oral, Frank Bogun

**Affiliations:** ^1^ Division of Cardiovascular Medicine, Department of Electrophysiology University of Michigan Ann Arbor Michigan USA

**Keywords:** bundle of his, catheter ablation, late gadolinium enhanced cardiac magnetic resonance, premature ventricular contraction

## Abstract

**Background:**

Cather ablation of parahisian premature ventricular complexes (PVCs) often requires ablation in multiple cardiac chambers, including the sinuses of Valsalva (SoV). The safety and efficacy of ablation within the right SoV to target parahisian arrhythmias has not been widely reported.

**Objective:**

To report on the demographic and procedural characteristics of patients undergoing catheter ablation of PVCs who underwent ablation in the right SoV, and to examine the impact of late‐gadolinium enhanced cardiac magnetic resonance (LGE‐CMR) on procedural findings.

**Methods:**

Consecutive patients undergoing ablation of parahisian PVCs and ablation in the right SoV with preprocedural LGE‐CMR were included.

**Results:**

Eleven patients were included in the study population (11 males (100%), median age: 68 ± 7 years, median ejection fraction: 53% ± 7%, PVC burden 23% ± 13%). Intramural LGE‐CMR scar was present in all patients and involved the basal anteroseptum/outflow tract in nine patients. Ablation within the right SoV eliminated (*n* = 9) or suppressed (*n* = 2) PVCs in all patients. The successful SoV site displayed the absolute earliest presystolic activation time or matching pacemaps in only 44% and 55% of patients, respectfully. Transient heart block during right SoV ablation occurred in 1/11(9%) patients. The post procedure PVC burden decreased from 23% ± 13% to 7% ± 6%, procedural success was attained in 10/11(91%) of patients.

**Conclusions:**

Parahisian PVCs ablated from the right SoV are often intramural, may require ablation in multiple chambers, and colocalize with intramural LGE‐CMR scar. Traditional EGM markers of successful ablation sites were less frequently seen at successful site of SoV ablation, long term success was achieved in 91% of patients.

## 1 Introduction

Catheter ablation is a safe and effective treatment for premature ventricular complexes (PVCs) [1]; however, the arrhythmia site of origin can greatly impact procedural outcomes. PVCs with a parahisian origin are challenging to target due to limitations in mapping within this region as well as the risk of collateral damage to the conduction system [2]. Parahisian PVCs often have intramural origins and may require mapping and ablation from multiple cardiac chambers. Ablation from above the aortic valve within the sinuses of Valsalva, specifically the right sinus of Valsalva, may be necessary to target parahisian PVCs, however, details of these procedures have not been adequately reported. The purpose of this is study is to examine the patient and procedural characteristics of patients undergoing ablation of parahisian PVCs requiring ablation from within the right sinus of Valsalva.

## 2 Methods

### 2.1 Patient Characteristics

Between 2017 and 2023, 604 patients underwent ablation of PVCs with preprocedure late gadolinium enhanced cardiac magnetic resonance imaging including 52 patients with parahisan PVCs. The final study population consisted of 11 consecutive patients (11 males (100%), median age: 68 ± 7 years, median ejection fraction: 53% ± 7%) undergoing cardiac ablation with preprocedure late‐gadolinium enhanced cardiac magnetic resonance imaging (LGE‐CMR) in whom the predominant ventricular arrhythmia was a parahisian PVC targeted with ablation from the sinus of Valsalva (Table 1). The preprocedure PVC burden was 23% ± 13%. A parahisian PVC was the predominant arrhythmia in all patients (mean number of morphologies 4 ± 3). Two patients had failed antiarrhythmic medications. No patients had cardiac electronic implantable devices. One patient underwent genetic testing revealing a variance of unknown significance in TNN13K. Patients who required ablation within the cusp were more likely to have LGE‐CMR scar (100% vs. 63%, *p* < 0.05) and had a trend towards wider PVC EKG width (163 ± 26 vs. 150 ± 17, *p* = 0.05) without other significant demographic, procedural, or EKG differences (Supporting Information S1: Table S1–S3). The study was approved by the University of Michigan Institutional Review Board.

**Table 1:** Patient characteristics.

	*n* = 11
Age, years (SD)	68.00 (7.30)
Sex, Male (%)	11 (100.0)
BMI, mean (SD)	29.86 (5.07)
PVC Burden Pre Ablation, % (SD)	23.36 (10.87)
EF Pre, % (SD)	53.45 (6.88)
CAD, *n* (%)	6 (54.5)
CABG, *n* (%)	1 (9.1)
HTN, *n* (%)	9 (81.8)
HLD, *n* (%)	9 (81.8)
DM, *n* (%)	3 (27.3)
AF, *n* (%)	5 (45.5)
PAD, *n* (%)	11 (100.0)
COPD, *n* (%)	11 (100.0)
Thyroid Disease, *n* (%)	1 (9.1)
CKD, *n* (%)	1 (9.1)
*Medication*	
Prior antiarrhythmic, *n* (%)	2 (20.0)
Beta blocker, *n* (%)	9 (81.8)
ACE inhibitor, *n* (%)	5 (45.5)
ARB, *n* (%)	1 (9.1)
Aldosterone antagonist, *n* (%)	2 (18.2)
Statin, *n* (%)	7 (63.6)
Antiplatelet, *n* (%)	3 (30.0)
NOAC, *n* (%)	1 (9.1)

Abbreviations: ACE, angiotensin converting enzyme; AF, atrial fibrillation; ARB, angiotensin receptor blocker; BMI, body mass index; CABG, coronary artery bypass graft; CAD, coronary artery disease; CKD, chronic kidney disease; COPD, chronic obstructive pulmonary disease; DM, diabetes mellitus; EF, ejection fraction; HLD, hyperlipidemia; HTN, hypertension; LGE‐CME, late gadolinium enhancement cardiac magnetic resonance; NOAC, novel oral anticoagulant; PAD, peripheral artery disease; PVC, premature ventricular contraction.

### 2.2 Electrophysiologic Study and Mapping

After informed consent was obtained, several multi‐polar electrode catheters were introduced into the right ventricle, and the His position. Venous and arterial access was attained, all patients underwent retrograde aortic access to access the cusps and left ventricular outflow tract. Heparin was administered to attain an ACT of > 250 s. In the case of infrequent PVC, isoproterenol infusion was initiated. Programmed right ventricular stimulation was performed using up to 4 extrastimuli to assess for inducible VT. If not inducible, programmed stimulation was repeated during infusion of isoproterenol at 2–4 µg/min. Electrograms were filtered at 50–500 Hz. The intracardiac electrograms and leads V1, I, II, and III were displayed on an oscilloscope and displayed at a speed of 100 mm/sec. The recordings were stored on optical disc (EP Med, West Berlin, New Jersey). An electroanatomical mapping system (CARTO, Biosense Webster, Diamond Bar, CA) was used for mapping. Activation mapping was performed during ventricular ectopy or ventricular tachycardia and entrainment mapping during VT if hemodynamically tolerated. In the setting of infrequent ventricular ectopy, pace‐mapping was used to identify the site of origin. Pace‐maps (output of 10 mv at 2 ms) were classified as matching (10/12 matching leads) or not matching. A parahisian origin was defined as a PVC with earliest endocardial mapping within 10 mm of any his potential identified on electroanatomic mapping [3]. An intramural SOO was defined as an arrhythmia in which pace mapping at the earliest site of activation failed to reproduce the same or similar QRS complex (10 > 12 matching leads) [4, 5, 6], and multiple chambers mapping resulting in similar (< 10 ms) activation times [7]. The right, left, and noncoronary sinuses of Valsalva were identified on intracardiac ultrasound and contours were integrated into the electroanatomic map using CartoSound (Biosense Webster, Diamond Bar, CA).

### 2.3 Catheter Ablation

Radiofrequency energy was delivered via a 3.5mm‐tip irrigated‐tip catheter (Smart Touch Surround flow, Thermocool or QDOT, Biosense Webster, Diamond Barr, CA). Target sites considered for ablation included sites with the earliest endocardial activation within that mapped chamber during the ventricular arrhythmia and/or at sites with matching pace‐mapping sites. All inducible VTs were targeted for ablation as well as predominant PVCs. Radiofrequency energy was delivered at a power of 30 Watts and titrated up to a maximum of 50 Watts. Radiofrequency energy was delivered for up to 120 s with a target impedance drop of 10–15 Ohms.

A stepwise approach was used beginning with radiofrequency energy with monitoring for junctional rhythm and discontinuing energy in the presence of PR prolongation or AV block. In these cases, focal cryoablation was performed at the discretion of the operating physician with an 6 mm tip catheter (Freezor Extra, Medtronic, Minneapolis, MN). Cooling was performed to −80℃ with a lesion duration of 180–240 s with discontinuation of ablation if there was evidence of conduction system block. After ablation a 30 min waiting period was observed; acute procedural success was defined as complete elimination of the clinical PVC, partial success was defined as reduction in the frequency without elimination, and failure was defined as no change in PVC frequency.

### 2.4 Cardiac Magnetic Resonance Imaging

LGE‐CMR studies were performed on a 1.5‐Tesla scanner (1.5 T Achieva Philips MR, Amsterdam, Netherlands) with an 8‐element phased array coil placed over the chest of patients in the supine position. Images were acquired with electrocardiogram gating during breath‐holds. Ten to 15 min after administration of 0.1–0.15 mmol/kg of intravenous gadobenate dimeglumine (MultiHance, Bracco Diagnostics, Princeton, NJ), 2‐D late gadolinium‐enhanced imaging was performed using an inversion‐recovery sequence [8] (repetition time 6.7 ms, echo time 3.2 ms, in‐plane spatial resolution 1.4 × 2.2 mm, slice thickness 8 mm) in the short‐axis and three long‐axis views of the left ventricle. The inversion time (250–350 ms) was optimized to null the normal myocardium in the short‐axis and long‐axis of the left ventricle at matching cine‐image slice locations.

### 2.5 Follow Up

Patients were seen in clinic 3 months postablation and followed for 12 months. Echocardiography and a 12 lead 24‐h Holter monitor were performed in all patients at the 3 month postablation follow up appointment. Follow‐up procedural success was defined as an 80% reduction in the preprocedure PVC burden [9].

### 2.6 Statistical Analysis

Continuous variables were expressed as mean ± 1 SD and were compared with the Student *t*‐test. Discrete variables were compared by chi‐square analysis or with the Fisher exact test. A *p* < .05 was considered statistically significant. Statistical analysis was performed using R version 4.2.2 (R foundation for Statistical Computing, Vienna, Austria).

## 3 Results

### 3.1 Cardiac Imaging

**Figure 1:**
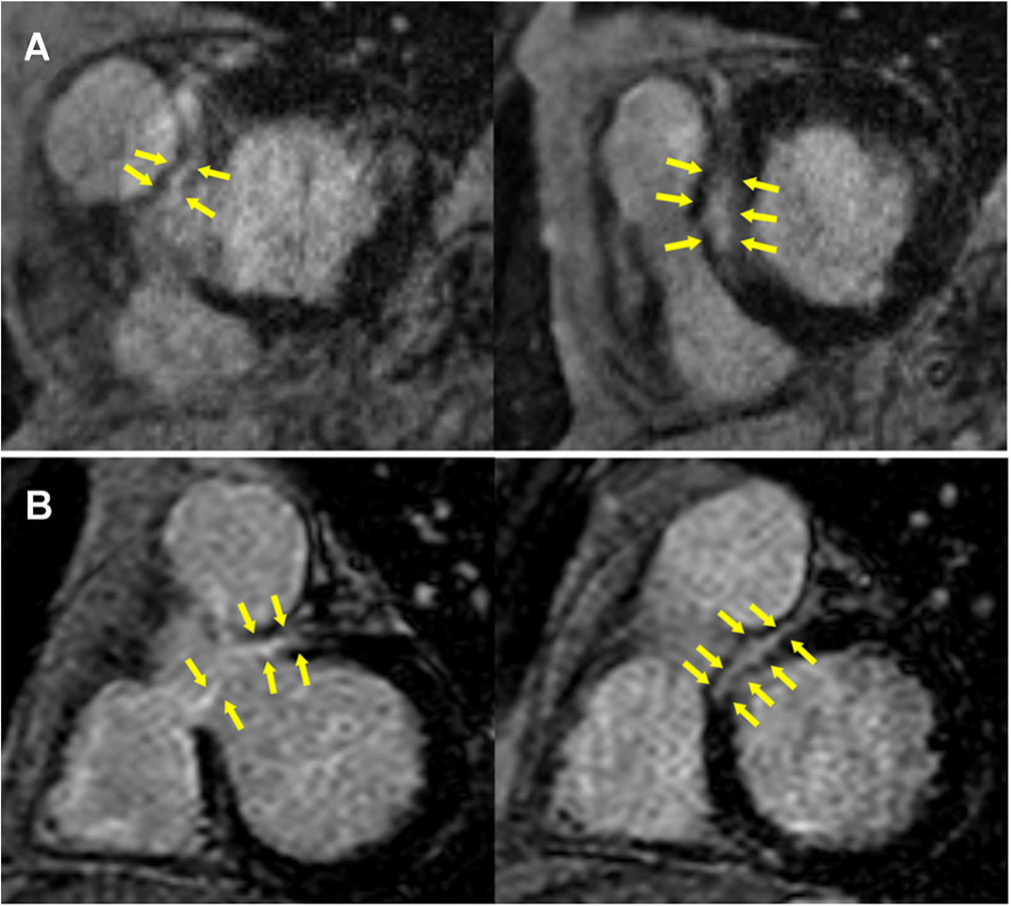
Preprocedure late gadolinium cardiac enhanced magnetic resonance imaging. Preprocedural imaging was obtained in all patients. Short axis slices are shown in two patients (Panel A and B) demonstrating intramural scar (yellow arrows) in the basal anteroseptal segments in the parahisian region extending toward the outflow tract. These patients had parahisian PVCs successfully ablated from within the sinus of Valsalva (Figures 2 and 3).

LGE‐CMR cardiac scar was present in all patients. The scar was located in the basal anteroseptal segments, extending into the LVOT adjacent to the right sinus of Valsalva in 9/11 patients (Figure 1). The remaining two patients had intramural basal inferoseptal scar and apical inferior scar. Basal scarring was intramural in all patients. Four‐patients with basal anteroseptal scar had multifocal intramural scar, with additional scar in the inferoseptal (*n* = 3), and inferolateral walls (*n* = 1). One patient with intramural basal anteroseptal scar had infarct related subendocardial scarring in the lateral segments.

### 3.2 Mapping and Ablation

All patients had parahisian PVCs (mean QRS width 163 ± 26 ms). All PVCs had a left bundle branch morphology, with dominant R wave in lead I, and axis was inferior in 9/11 patients. The PVC precordial lead transition occurred earlier than V4 in 9/11 patients, and was earlier than the native QRS precordial lead transition in 9/11 patients (Table 2). All patients underwent mapping within the right ventricle, left ventricle, and the sinuses of Valsalva. Two patients underwent mapping within the coronary venous system and five underwent mapping of the right atrium/posterior superior process. The earliest presystolic bipolar EGMs recorded within the right ventricle were −19 ± 10 ms, left ventricle −28 ± 15 ms, and within the sinus of Valsalva −21 ± 11 ms. No patient had matching pacemaps from with the right ventricle. Two patients had matching pacemaps from the left ventricle. Four patients had matching pacemaps from with the right sinus of Valsalva, while two patient had noncapture despite high‐output pacing from the right sinus of Valsalva.

**Table 2:** EKG characteristics.

	*n* = 11
PVC morphology change with ablation, *n* (%)	3 (27.3)
PVC width, ms (SD)	163.27 (26.42)
Lead I, mV (ms)	1.18 (0.76)
Lead II, mV (ms)	0.92 (0.37)
Lead III, mV (ms)	−0.37 (1.12)
Lead I positive, *n* (%)	10 (90.9)
Lead II/III discordant, *n* (%)	6 (54.5)
PVC precordial transition	
V2	5 (45.5)
V3	4 (36.4)
V4	1 (9.1)
V5	0(0)
V6	1 (9.1)
Native QRS precordial transition	
V2	1 (9.1)
V3	1 (9.1)
V4	3 (27.3)
V5	2 (18.2)
V6	4 (36.4)
PVC precordial transition earlier than native QRS, *n* (%)	9 (81.8)

Abbreviations: LBIA, left bundle inferior axis; LBSA, left bundle superior axis; LGE‐CMR, late gadolinium enhanced cardiac magnetic resonance; LV, left ventricle; ms, milliseconds; mv, millivolts; PVC, premature ventricular contraction; RBIA, right bundle inferior axis; RBSA, right bundle superior axis; RV, right ventricle.

**Figure 2:**
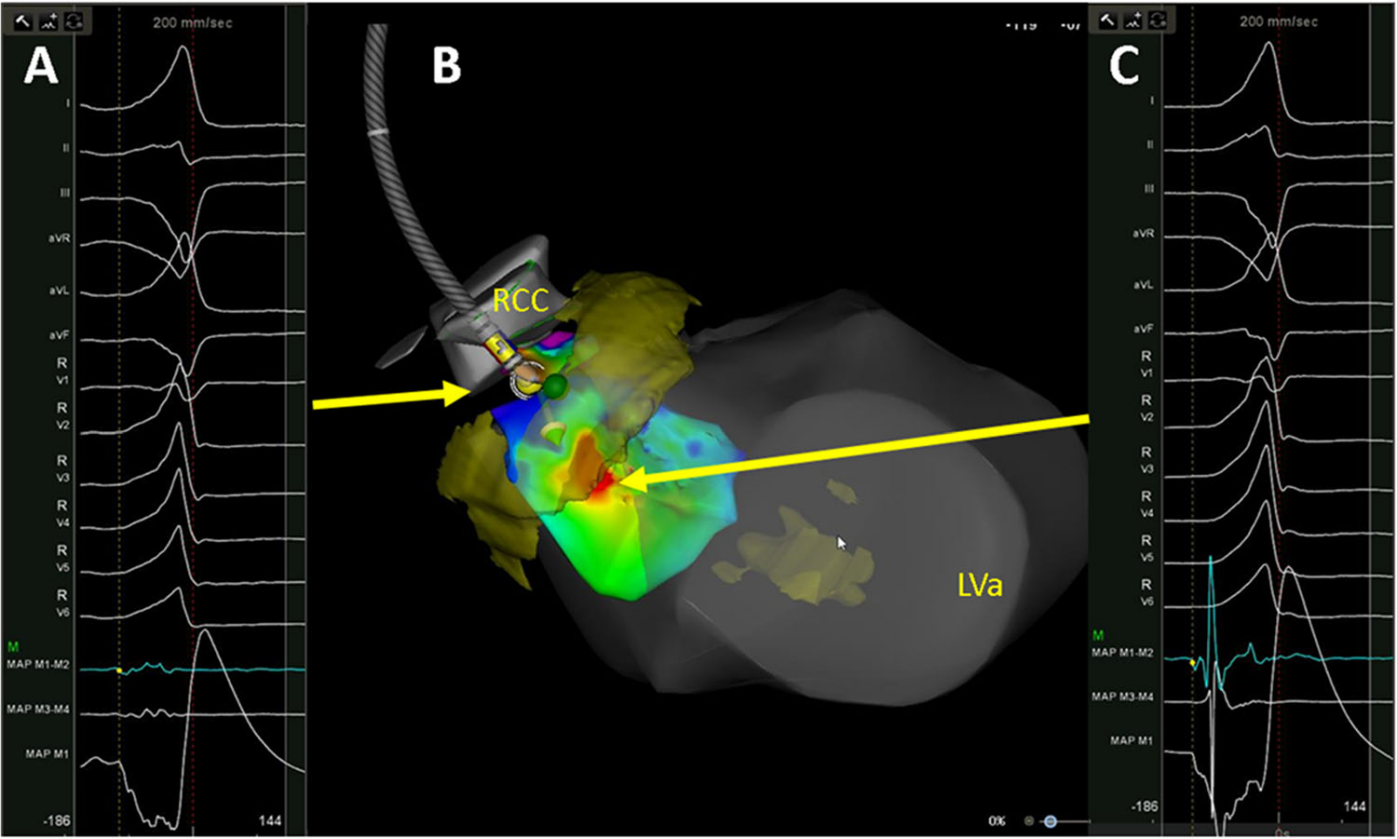
Ablation of parahisian premature ventricular complex. Electroanatomic map with activation data taken from within the left ventricle and right sinus of Valsalva is shown, three‐dimensional reconstruction and overlay of the late‐gadolinium enhanced cardiac magnetic resonance imaging (Figure 1A) is shown in yellow (Pane B). Isochronal maps are shown with early sites of activation shown in red near the parahisian region, located within 10 mm of the his cloud. Presystolic signals from the left ventricle (Panel C) are shown, pace maps in this region were poor and ablation here had no impact on the clinical PVC and resulted in junctional tachycardia. Within the RCC (Panel A), far‐field, low voltage signals at the site of the catheter position shown in Panel B demonstrated similar presystolic timing. There was noncapture in this areas; however, ablation here and at yellow tags resulted in the elimination of the PVC. RCC, right coronary cusp. LVa, left ventricular apex.

**Figure 3:**
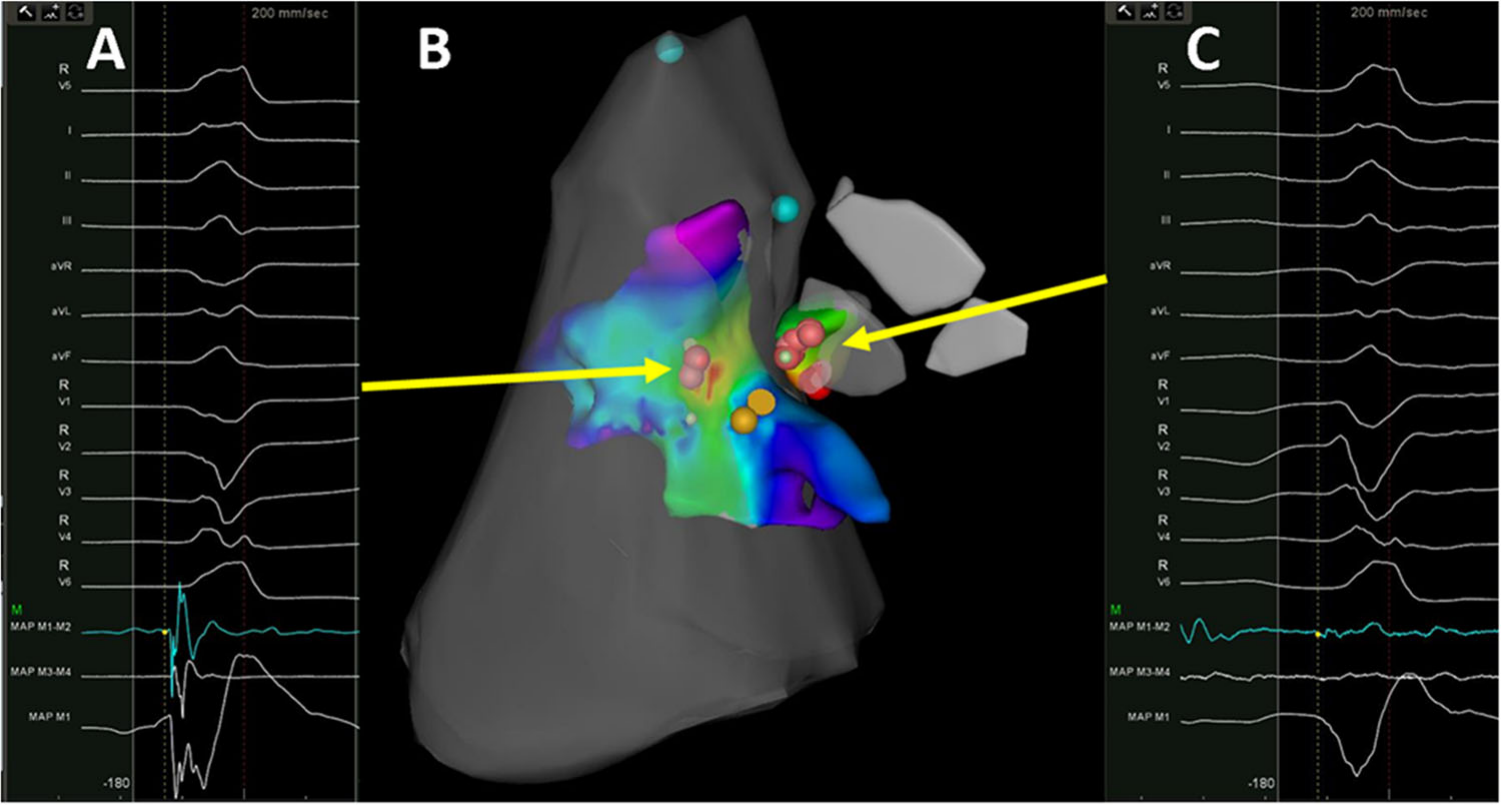
Ablation of parahisian premature ventricular complex. Electroanatomic map with activation data taken from within the right ventricle and right sinus of Valsalva is shown (Panel B). Isochronal maps are shown with early sites of activation in the right ventricle near the his cloud (yellow tags). Pace maps were poor in this region; ablation in the right ventricle (red tags) resulted in partial suppression of the PVC and temporary heart block which recovered after cessation of ablation. Mapping within the right sinus of Valsalva was performed, demonstrating far‐field, low‐voltage signals (Panel C). Despite proximity to the his cloud (4 mm), ablation here resulted in elimination of the premature ventricular complex without conduction disturbances. Preprocedure late gadolinium enhanced cardiac magnetic resonance demonstrated multifocal scar, including intramural basal anteroseptal scar near the arrhythmia site of origin (Figure 1 Panel B).

Patients underwent RF ablation alone (*n* = 7) or with additional cyroablation (*n* = 4). Ablation was performed within the right ventricle in nine patients, and left ventricle in 7 (64%) patients. Ablation in these chambers resulted in change in the PVC morphology without decrease in frequency in 3 (27%) patients. During ablation in the right or left ventricle, patients developed conduction disturbances during ablation included junctional rhythm (*n* = 5), transient right‐bundle bundle branch block (*n* = 4), and transient complete heart block (*n* = 4) (Table 3). Radiofrequency ablation was performed in all patients within the right sinus of Valsalva (Figure 2 and Figure 3). Within the cusps patients underwent 7 ± 4 lesions with an average starting power of 29.5 ± 4.7 watts and duration of 93 ± 13 s. A target site within the RCC demonstrated a far‐field his in four patients, ablation was performed at the nearest adjacent site without a his in these patients. Transient heart block was noted during radiofrequency ablation from the right sinus of Valsalva in one patient, and the patient subsequently underwent cryoablation within the right sinus of Valsalva without conduction disturbances. No His signal was seen at the site of heart block in this patient. At the earliest site of activation, the bipolar EGMs were 0.4 ± 0.2 mv and 60 ± 13 ms. Ablation from the right sinus of Valsalva was successful in eliminating the PVC in 9 patients (82%) and resulted in suppression without elimination in 2 (18%). Among 9 patients with successful ablation at the sinus of valsalva, a matching pacemap to the clinical PVC was seen in 4/9 (44%) patients, and the sinus of valsalva bipolar EGMs were earlier than right/left ventricular EGMs in 5/9 (55%) of patients. A qs complex on the unipolar EGM was noted in all patients with successful ablation site from the right sinus of Valsalva. Acute procedural success was attained in 9/11 (82%) of patients. All patients underwent programmed ventricular stimulation. One patient was found to be inducible for sustained monomorphic ventricular tachycardia which was targeted and successfully ablated.

**Table 3:** Procedural characteristics.

	*n* = 11
PVC burden preablation, % (SD)	23.36 (10.87)
PVC burden postablation, % (SD)	7.64 (11.49)
Inducible VT, *n* (%)	1 (9.1)
Multiple sites ablated, *n* (%)	11 (100.0)
PVC change in morphology with ablation, *n* (%)	3 (27.3)
RFA time, min (SD)	25.73 (7.42)
Procedure time, min (SD)	236.10 (68.30)
Fluroscopy time, min (SD)	21.44 (11.86)
Conduction disturbances	
None	3 (27.3)
Transient heart block	4 (36.4)
New persistent bundle branch block	4 (36.4)
Junctional rhythm with ablation, *n* (%)	5 (45.5)

Abbreviations: LGE‐CME, late gadolininum enhancement cardiac magnetic; LVOT, left ventricular outflow tract; PVC, premature ventricular contraction; RFA, radiofrequency ablation; RVOT, right ventricular outflow tract; VT, ventricular tachycardia.

All 9 patients with basal anterior intramural scar had evidence of intramural PVCS. Patients with septal scar had lower ejection fractions that those without (51% ± 6% vs. 62% ± 3%, *p* = 0.03) without other significant patient or procedural differences (*p* > 0.05). Patients without septal scar had earlier local bipolar EGMS within the LV (−46.50 ± 19.09 vs. −22.75 ± 10.02 ms, *p* = 0.03) without other significant EGM differences in other chambers. One patient without basal scar demonstrated similar activation time within the RV (−30 ms), LV (−33), and right sinus of Valsalva (−25 ms) suggesting intramural origin. Ablation from the the LV resulted in transient heart block, and successful ablation was performed from the right sinus of Valsalva. The remaining patient without scar demonstrated earliest activation within the LV (−60 ms) compared to the RV (−25 ms) or right sinus of Valsalva (−10 ms) without matching pacemaps in any cardiac chamber. Ablation at the LV led to partial suppression, with elimination after ablation from the right sinus of Valsalva.

### 3.3 Complications and Follow Up

There were no major procedural complications. Conduction system remained intact in all patients postablation with no significant changes in PR intervals or QRS morphology or duration. One patient with inducible ventricular tachycardia underwent an uncomplicated implantable cardiac defibrillator placement before discharge. No patient underwent a repeat ablation procedure, the PVC burden decreased from 23% ± 13% to 7% ± 6%, all patients but one had at least 80% reduction in PVC burden at follow up, including those with only partial suppression with sinus of Valsalva ablation. No patient developed sustained ventricular arrhythmias over a follow up 11 IQR [6–22] months. There were no instances of aortic valve injury on follow up transthoracic echocardiogram.

## 4 Discussion

The right sinus of Valsalva can be a useful vantage point from which ventricular arrhythmias originating from the parahisian region can be targeted safely and effectively via an anatomic approach. The majority of patients with parahisian PVCs successfully ablated from the right sinus of Valsalva demonstrated evidence of intramural cardiac scar on LGE‐CMR in the basal anteroseptal region. Transient conduction abnormalities during ablation within the sinus of Valsalva were infrequent; no patients developed permanent heart block and long‐term procedural success was achieved in 91% of patients.

**Figure 4:**
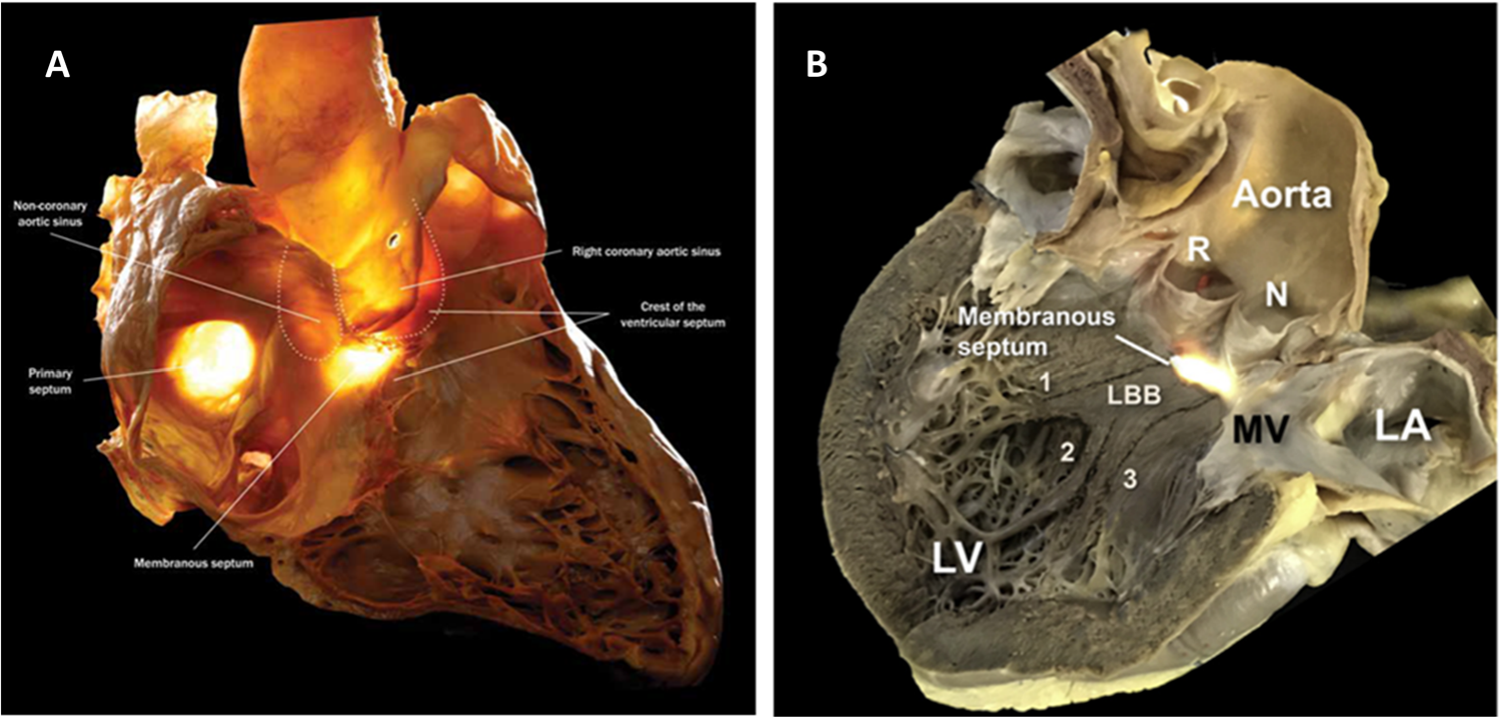
Anatomic relationship of the sinus and Valsalva and the cardiac conduction system. Panel A: Transillumination of a prepared heart displayed in the right anterior oblique view is shown. Dissection has been performed to reveal a sectional plane of mid‐line structures. The penetrating bundle and nonbranching sections of the bundle of his extend through the membranous septum and crest of the ventricular septum, which lay in close proximity to the non and right sinuses of Valsalva. Panel B: Transillumination of a second prepared heart is displayed in the left posterior oblique view. Dissection has been performed to reveal midline structures. The endocardial portion of the left bundle branch along with anterior (1), septal (2), and posterior (3) fascicles are shown inferior to the membraneous septum in close proximity to sinus of valsalva. R, right coronary sinus. N, noncoronary sinus. LA, left atrium. LV, left ventricle. MV, mitral valve. Panel A used with permission from Mori et al. [10] (https://creativecommons.org/licenses/by-nc-nd/4.0/). Panel B used and modified with permission from Cabera et al. [11] (https://creativecommons.org/licenses/by-nc-nd/4.0/).

The sinus of Valsalva is anatomically related to multiple components of the conduction system, and provides opportunities for mapping and ablation of parahisian arrhythmias. The compact atrioventricular node is located at the apex of triangle of Koch which is formed by the membranous septum and which itself is located inferiorly to the right and noncoronary sinuses of Valsalva (Figure 4A). The specialized conduction tissue continues to become the penetrating bundle of His, running a variable course through the membranous septum [12]; given this relationship it is not surprising that parahisan arrhythmias may be ablated from the atrial slow‐pathway region [2]. The branching portion of the his bundle, and arborization of the left bundle system also occurs just inferior to the right and noncoronary sinuses of Valsalva (Figure 4B). The sinus of valsalva is separated from the ventricular myocardium by fibrous structures composing the cardiac skeleton including the right fibrous trigone which is in continuity with the membranous septum. These structures may explain the far‐field and low‐voltage signals encountered at successful sites of ablation within the sinus of Valsalva. Given the variable course of the his bundle in this region and the attenuated EGMS signals, heart block during ablation may occur even in the absence of a His signal on the local EGMs, as was encountered in one patient in this series.

In this series, the majority of patients with parahisian PVCs ablated from with the right sinus of Valsalva were found to have intramural cardiac scar near the site of origin. This is similar to prior reports demonstrating that LGE‐CMR scar colocalizes with the predominant PVC site of origin in 90% undergoing ablation procedures [13]. Electroanatomic mapping data was also consistent with intramural PVCs with similar activation times in multiple chambers. A QS signal on the unipolar catheter was seen in all patients within the cusp at successful sites. In contrast, Sabzwari et al reported a QS unipolar signal at successful ablation sites in only 53% of patients undergoing right and left outflow tract ablation for intramural PVCs [14]. These differences may be due to the predominance of fibrous tissue rather than myocardial tissue encountered when mapping within the cusp as opposed to the ventricular outflow tract. Future studies with high‐density mapping catheters may helpful. Additionally, change in PVC morphology without reduction in PVC burden was seen in 28% of patients, consistent with prior studies on ablation of intramural arrhythmias [15]. The sinuses of Valsalva are a common site of ablation in patients with periaortic scar [16], and basal ventricular arrhythmias [17, 18] and this series extends these findings to patients with parahisian arrhythmias. The precise site of origin of intramural arrhythmias can be difficult to ascertain without the use of intramural mapping techniques such as wire‐mapping within perforator veins [4], which are often unaaccessible near the parahisian region [19]. The site of origin may be approximated by endocardial activation time; however, due to preferential conduction in the basal and periaortic regions, the earliest site of electrical activation may be remote from the anatomic site of origin [20]. A previous study of ablation of patients with parahisian arrhythmias from the aortic sinuses of Valsalva demonstrated similar activation times with the endocardium and aortic sinus of Valsalva [21]. Similarly, in this series many patients with successful ablation within the sinus of Valsalva failed to display EGM characteristics of traditional target sites such as matching pacemaps or demonstrating the absolute earliest bipolar EGM time, which was seen even in the absence of LGE‐CMR scar. While further studies are needed to define adequate target sites within this structure, ablation at the earliest site of activation within the sinus of Valsalva was effective in eliminating the clinical PVC in the majority of patients.

### 4.1 Clinical Implications

Parahisian PVCs are often intramural and require mapping and ablation from multiple cardiac chambers including the right sinus of valsalva. Preprocedural LGE‐CMR can help identify the PVC site of origin, including the intramural nature of the arrhythmia. The anatomic and electrical complexities of this region, proximity to the conduction system, and the challenges of mapping in the presence of intramural scar may render traditional targets of ablation less reliable. Nevertheless, ablation at the earliest site within the sinus of Valsalva, with close monitoring for conduction disturbances, successfully eliminated the PVC in the majority of patients. While prior studies have reported successful parahisian PVC ablation from the noncoronary sinus, the right coronary sinus was most successful site of ablation in this series. Preprocedure demographic data among patients with parahisian PVCs requiring ablation within the cusps were similar to those not requiring ablation within the cusps; detailed mapping in all chambers, including the cusps, is critical for success among patients with parahisian PVCs.

### 4.2 Limitations

This was a single‐center study and the results should be confirmed with larger multicenter investigations. This was a retrospective series; a predefined mapping or ablation approach was not used. Multipolar mapping catheters and vendor‐specific quantification of pacemaps were not used. Visual assessment of pacemapping has known limitations. The mean QRS duration of parahisian PVCs was wider than reported in other series, larger comparative series are required to understand the validity and significance of this observation. All patients in this series were found to have intramural LGE‐scar, usually located in the parahisian region; larger comparison to patients without cardiac scar requiring ablation within the sinus of Valsalva may yield better insights into the optimal mapping and ablation techniques. Quantitative analysis of LGE‐CMR parameters was not performed. All patients in this consecutive series were male, larger series are needed to understand if this is reflective of local populations and practice biases or by physiological differences between sexes.

## 5 Conclusion

Parahisian arrhythmias often have intramural origins and in many instances may be eliminated safely with ablation from the right sinus of Valsalva. Close monitoring for conduction disturbances is required as heart block may occur even in the absence of local His signals. Consistent with other intramural arrhythmias, ablation within multiple chambers may be necessary, and traditional EGM markers of successful ablation sites are less reliable when targeting parahisian arrhythmias; however, ablation at early sites within the right sinus of Valsalva was successful in the majority of patients.

## Disclosure

The authors have nothing to report.

## Supporting information

Supporting information.

## Data Availability

Research data are not shared.
