# Supplementary material for: Catheter Ablation of Parahisian Premature Ventricular Complexes From the Right Sinus of Valsalva
Source: J Cardiovasc Electrophysiol. 2024 Dec 8;36(4):731–8. doi: 10.1111/jce.16513 (PMC11984338; doi:10.1111/jce.16513)
Supplement: Supplementary file 1 — Supporting information. [file JCE-36-731-s001.docx]

**Supplemental Table 1**

|  | Parahisian PVC (n=52) | No Cusp Ablation  (n=41) | Cusp Ablation (n=11) | P |
| --- | --- | --- | --- | --- |
| Age, yrs(sd) | 66.24 (10.21) | 65.79 (10.86) | 68.00 (7.30) | 0.548 |
| Sex, Male(%) | 42 ( 84.0) | 31 ( 79.5) | 11 (100.0) | 0.241 |
| BMI, mean(sd) | 30.10 (4.78) | 30.15 (4.79) | 29.86 (5.07) | 0.881 |
| PVC Burden Pre Ablation, %(sd) | 20.71 (11.40) | 20.00 (11.56) | 23.36 (10.87) | 0.39 |
| EF Pre, %(sd) | 50.44 (11.36) | 49.63 (12.23) | 53.45 (6.88) | 0.327 |
| CAD, n(%) | 21 ( 42.0) | 15 ( 38.5) | 6 ( 54.5) | 0.543 |
| CABG, n(%) | 2 ( 4.0) | 1 ( 2.6) | 1 ( 9.1) | 0.917 |
| HTN, n(%) | 35 ( 70.0) | 26 ( 66.7) | 9 ( 81.8) | 0.551 |
| HLD, n(%) | 34 ( 68.0) | 25 ( 64.1) | 9 ( 81.8) | 0.455 |
| DM, n(%) | 13 ( 26.0) | 10 ( 25.6) | 3 ( 27.3) | 1 |
| AF, n(%) | 14 ( 28.0) | 9 ( 23.1) | 5 ( 45.5) | 0.28 |
| PAD, n(%) | 50 (100.0) | 39 (100.0) | 11 (100.0) | NA |
| COPD, n(%) | 8 ( 16.0) | 8 ( 20.5) | 0 ( 0.0) | 0.241 |
| Thyroid Disease, n(%) | 7 ( 14.0) | 6 ( 15.4) | 1 ( 9.1) | 0.969 |
| CKD, n(%) | 4 ( 8.0) | 3 ( 7.7) | 1 ( 9.1) | 1 |
| *Medication* |  |  |  |  |
| Prior Antiarrhythmic, n(%) | 14 ( 28.6) | 12 ( 30.8) | 2 ( 20.0) | 0.779 |
| Beta Blocker, n(%) | 38 ( 74.5) | 29 ( 72.5) | 9 ( 81.8) | 0.812 |
| ACE Inhibitor, n(%) | 23 ( 46.0) | 18 ( 46.2) | 5 ( 45.5) | 1 |
| ARB, n(%) | 10 ( 20.4) | 9 ( 23.7) | 1 ( 9.1) | 0.527 |
| Aldosterone Antagonist, n(%) | 6 ( 12.0) | 4 ( 10.3) | 2 ( 18.2) | 0.85 |
| Statin, n(%) | 30 ( 61.2) | 23 ( 60.5) | 7 ( 63.6) | 1 |
| Antiplatelet, n(%) | 14 ( 27.5) | 11 ( 26.8) | 3 ( 30.0) | 1 |
| NOAC, n(%) | 8 ( 15.4) | 7 ( 17.1) | 1 ( 9.1) | 0.856 |

**Supplemental Table 1**) Patient Characteristics. LGE-CME late gadolinium enhancement cardiac magnetic resonance, BMI body mass index, PVC premature ventricular contraction, EF ejection fraction, CAD coronary artery disease, CABG coronary artery bypass graft, HTN hypertension, HLD hyperlipidemia, DM diabetes mellitus, AF atrial fibrillation, PAD peripheral artery disease, COPD chronic obstructive pulmonary disease, CKD chronic kidney disease, ACE angiotensin converting enzyme, ARB angiotensin receptor blocker, NOAC novel oral anticoagulant

**Supplemental Table 2**

|  | Parahisian PVC (n=52) | No Cusp Ablation  (n=41) | Cusp Ablation (n=11) | P |
| --- | --- | --- | --- | --- |
| PVC Morphology, n(%) |  |  |  | 0.163 |
| RBIA | 2 ( 3.8) | 2 ( 4.9) | 0 ( 0.0) |  |
| RBSA | 1 ( 1.9) | 1 ( 2.4) | 0 ( 0.0) |  |
| LBIA | 27 (51.9) | 18 (43.9) | 9 ( 81.8) |  |
| LBSA | 22 (42.3) | 20 (48.8) | 2 ( 18.2) |  |
| LGE-CRM Scar, n(%) | 37 (71.2) | 26 (63.4) | 11 (100.0) | 0.045 |
| PVC Morphology Change with Ablation, n(%) | 11 (21.2) | 8 (19.5) | 3 ( 27.3) | 0.886 |
| PVC Width, ms(sd) | 152.81 (20.07) | 150.00 (17.34) | 163.27 (26.42) | 0.05 |
| Lead I, mV(ms) | 0.83 (0.84) | 0.74 (0.85) | 1.18 (0.76) | 0.123 |
| Lead II, mV(ms) | 0.58 (0.63) | 0.49 (0.65) | 0.92 (0.37) | 0.046 |
| Lead III, mV(ms) | -0.55 (0.94) | -0.60 (0.90) | -0.37 (1.12) | 0.469 |
| Lead I Positive, n(%) | 44 (84.6) | 34 (82.9) | 10 ( 90.9) | 0.856 |
| Lead II/III Discordant, n(%) | 34 (65.4) | 28 (68.3) | 6 ( 54.5) | 0.621 |
| PVC Precordial Transition |  |  |  | 0.3 |
| V2 | 15 (28.8) | 10 (24.4) | 5 ( 45.5) |  |
| V3 | 13 (25.0) | 9 (22.0) | 4 ( 36.4) |  |
| V4 | 10 (19.2) | 9 (22.0) | 1 ( 9.1) |  |
| V5 | 7 (13.5) | 7 (17.1) | 0 ( 0.0) |  |
| V6 | 7 (13.5) | 6 (14.6) | 1 ( 9.1) |  |
| Native QRS Precordial Transition |  |  |  | 0.72 |
| V2 | 8 (15.4) | 7 (17.1) | 1 ( 9.1) |  |
| V3 | 5 ( 9.6) | 4 ( 9.8) | 1 ( 9.1) |  |
| V4 | 12 (23.1) | 9 (22.0) | 3 ( 27.3) |  |
| V5 | 15 (28.8) | 13 (31.7) | 2 ( 18.2) |  |
| V6 | 12 (23.1) | 8 (19.5) | 4 ( 36.4) |  |
| PVC Precordial Transition Earlier than Native QRS, n(%) | 30 (57.7) | 21 (51.2) | 9 ( 81.8) | 0.139 |
|  |  |  |  |  |

**Supplemental Table 2)** EKG Characteristics. RV Right ventricle, LV left ventricle, RBIA right bundle inferior axis, RBSA right bundle superior axis, LBIA left bundle inferior axis, LBSA left bundle superior axis, PVC premature ventricular contraction, LGE-CMR late gadolinium enhanced cardiac magnetic resonance, ms milliseconds, mv millivolts

**Supplemental Table 3)**

|  | Parahisian PVC (n=52) | No Cusp Ablation  (n=41) | Cusp Ablation (n=11) | P |
| --- | --- | --- | --- | --- |
| PVC Burden Pre Ablation, %(sd) | 20.71 (11.40) | 20.00 (11.56) | 23.36 (10.87) | 0.39 |
| PVC Burden Post Ablation, %(sd) | 5.65 (7.95) | 5.34 (7.81) | 6.73 (8.74) | 0.616 |
| Inducible VT, n(%) | 6(11.5) | 5(12.2) | 1(9.1) | 0.83 |
| PVC Change in Morphology with Ablation, n(%) | 11 (21.2) | 8 (19.5) | 3 ( 27.3) | 0.886 |
| RFA time, min(sd) | 26.75 (18.18) | 27.05 (20.39) | 25.73 (7.42) | 0.834 |
| Procedure time, min(sd) | 252.71 (81.31) | 257.08 (84.68) | 236.10 (68.30) | 0.474 |
| Fluroscopy time, min(sd) | 19.61 (13.56) | 19.16 (14.06) | 21.44 (11.86) | 0.656 |
| Conduction Disturbances |  |  |  |  |
| None | 28 (53.8) | 25 (61.0) | 3 ( 27.3) | 0.022 |
| Transient Heart Block | 17 (32.7) | 13 (31.7) | 4 ( 36.4) |  |
| New Persistent Bundle Branch Block | 6 (11.5) | 2 ( 4.9) | 4 ( 36.4) |  |
| Junctional Rhythm with Ablation, n(%) | 22 (42.3) | 17 (41.5) | 5 ( 45.5) | 1 |

**Supplemental Table 3**) Procedural Characteristics. PVC premature ventricular contraction, RVOT right ventricular outflow tract, LVOT left ventricular outflowtract, VT ventricular tachycardia, RFA radiofrequency ablation.
